# Dr. James A. S. Grant (Grant-Bey)

**Published:** 1896-09

**Authors:** 


Dr. James A. S. Grant (Grant-Bey) died at the Bridge of Allan, Scotland, July 28, 1896. He had been in delicate health for some time, and left his residence at Cairo, Egypt, for treatment at a German Spa, on account of a troublesome heart affection. Not finding the expected relief, he went to Scotland, where he was born, and where he tried the local springs, but with no better results. He reached his native country a short time before his death.

Dr. Grant was born at Meyhill, Scotland, about the year 1840. He was graduated at the University of Aberdeen, and went to Alexandria, Egypt, in 1865. Shortly afterward he went to Cairo, where he had since resided. His skill in his profession soon brought him distinction, and he became court physician with the title of Grant-Bey. He married Miss Ada M. Torrey, daughter of the late John Torrey, of Honesdale, Pa., February 7, 1870. She died in 1886, leaving two children, William Torrey Grant, now in Edinburgh University, and Jessie Campbell Grant. In the summer of 1890, Dr. Grant married Miss Florence Gibson, of Liverpool, England, who survives him with three children, one son and two daughters.

Dr. Grant-Bey was well known in this country, and a few years ago attended and read a paper at the annual meeting of the medical society of the State of New York.

